# Ulyptol

**Published:** 1895-08

**Authors:** 


### Ulyptol.

Ulyptol belongs in the same category with steresol. It is occasionally mentioned as a "new antiseptic." It was originally named and introduced in 1886, and is prepared by mixing six parts salicylic acid, one part carbolic acid and one part oil eucalyptus. It is also known as eulyptol, and the mixture is of service in treating wounds—*American Therapist.*

Nitroglycerine, three drops a day of a one per cent. solution, is a powerful anti-neuralgic, especially in persistent sciatica.

